# Effect of gut microbiome regulated Taohong Siwu Decoction metabolism on glioma cell phenotype

**DOI:** 10.3389/fcimb.2023.1192589

**Published:** 2023-06-05

**Authors:** Suyin Feng, Quan Wan, Weijiang Wu, Chenyang Zhang, Hua Lu, Xiaojie Lu

**Affiliations:** ^1^ Department of Neurosurgery, Affiliated Hospital of Jiangnan University, Wuxi, China; ^2^ Neuroscience Center, Wuxi School of Medicine, Jiangnan University, Wuxi, China; ^3^ Jiangnan University Medical Center, Wuxi, China; ^4^ Institute of Integrative Chinese and Western Medicine, Affiliated Hospital of Jiangnan University, Wuxi, China; ^5^ Wuxi Neurological Institute, Wuxi No.2 People’s Hospital, Wuxi, China

**Keywords:** Taohong Siwu Decoction, intestinal flora, CDC6 pathway, glioma cells, RNA-seq analysis

## Abstract

**Introduction:**

To establish a new model for exploring the mechanism of the gut microbiome and drug metabolism, we explored whether Taohong Siwu Decoction acts after metabolism by intestinal flora under the premise of clarifying the interaction between intestinal flora and drug metabolism.

**Methods:**

Taohong Siwu Decoction (TSD) was fed to germ-free mice and conventional mice, respectively. The serum from both groups of mice was removed and co-cultured with glioma cells in vitro. The co-cultured glioma cells were compared separately for changes at the RNA level using RNA-seq technology. The genes of interest in the comparison results were selected for validation.

**Results:**

The differences in the phenotypic alterations of glioma cells between serum from TSD-fed germ-free mice and normal mice were statistically significant. *In vitro* experiments showed that Taohong Siwu Decoction-fed normal mouse serum-stimulated glioma cells, which inhibited proliferation and increased autophagy. RNA-seq analysis showed that TSD-fed normal mouse serum could regulate CDC6 pathway activity in glioma cells. The therapeutic effect of TSD is significantly influenced by intestinal flora.

**Conclusion:**

The treatment of tumors by TSD may be modulated by intestinal flora. We established a new method to quantify the relationship between intestinal flora and the regulation of TSD efficacy through this study.

## Introduction

The gut microbiome, or intestinal flora, can have a significant impact on the metabolism of traditional Chinese medicine (TCM) compounds (Lin et al., 2021). The gut microbiome consists of trillions of microorganisms that reside in the human digestive tract, and it plays an important role in the breakdown and absorption of nutrients and drugs (Zhang et al., 2022). Research has shown that the gut microbiome can modify the pharmacokinetics and bioavailability of TCM compounds (Xia et al., 2022). This can impact the metabolism of TCM compounds by altering the way they are absorbed, distributed, metabolized, and eliminated from the body. For instance, the gut microbiome affects the activity of enzymes involved in the metabolism of TCM compounds, such as cytochrome P450 enzymes (Boer et al., 2016; Tang et al., 2022). Also, intestinal flora alters the expression of transporters that are responsible for moving TCM compounds across cell membranes, which can affect their absorption and bioavailability (Taguer et al., 2021; Togao et al., 2021). Furthermore, the gut microbiome produces metabolites that interacts with TCM compounds, potentially affecting their efficacy and safety (Chen et al., 2021; Yang and Wu, 2023). Some of these metabolites have anti-inflammatory or anti-tumor effects, which could be beneficial for TCM treatments for cancer (Feng et al., 2019; Gong et al., 2020). Overall, the gut microbiome is an important factor to consider when studying the metabolism of TCM compounds, as it can have significant implications for their efficacy and safety.

A growing number of studies have confirmed the important role of the gut microbiome in drug metabolism. Vijay found that gut microbes could significantly affect the pharmacokinetics of the drug digoxin, a medication used to treat heart failure (Vijay and Valdes, 2022). The researchers found that certain gut bacteria could alter the expression of genes involved in digoxin metabolism, leading to variations in drug levels and potential toxicity. Another investigation showed that the gut microbiome could influence the efficacy of the chemotherapy drug cyclophosphamide (Alexander et al., 2017). Alexander believed that certain bacteria could activate the drug by converting it into its active form, while others could deactivate it by breaking it down into inactive metabolites. Makki investigated the effect of the gut microbiome on the metabolism of the antidepressant drug fluoxetine (Makki et al., 2018). The researchers found that certain gut bacteria could metabolize the drug into inactive compounds, potentially reducing its efficacy. Furthermore, Weersma summarized the current state of knowledge on the role of the gut microbiome in drug metabolism (Weersma et al., 2020). He concluded that the gut microbiome can affect the pharmacokinetics and pharmacodynamics of a wide range of drugs, including TCM compounds. These studies suggest that the gut microbiome can play a significant role in drug metabolism, and that understanding this relationship could have important implications for drug development and personalized medicine.

Previous studies have clarified the correlation between drug metabolism and intestinal flora. However, it is unclear how to conduct quantitative studies, how to design drugs based on this correlation, and how to use this correlation in clinical treatment for the benefit of patients. The study of these complex issues urgently requires a quantitative model to explore the molecular mechanisms behind the correlations. How to identify *in vitro* whether the intestinal flora is directly involved in the regulation of drug metabolism is a difficult task. Our study attempts to obtain serum rich in drug metabolites through germ-free animals. Based on the serum from sterile animals, we determined whether intestinal flora is involved in drug metabolism.

## Materials and methods

### Materials

Twelve 10-12 weeks old, C57 mice; Fifteen 4 weeks female nude mice; DMEM/F-12 and fetal bovine serum (FBS) were from Thermo Fisher (Shanghai, China).

### Methods

#### Herb preparation

All Chinese herbs in Taohong Siwu Decoction (TSD) contain Prunus davidiana (CarriŠre) Franch. (family Rosaceae), Angelica sinensis (Oliv.) Diels (family Apiaceae), Carthamus tinctorius L. (family Asteraceae), Ligusticum striatum DC. (family Apiaceae), Rehmannia glutinosa (Gaertn.) DC. (family Orobanchaceae), and Paeonia delavayi Franch to facilitate repeated quality control. Based on the body surface area of mice, equivalent doses were calculated. All herbs (3:3:2:4:3) were mixed and diluted with water to prepare the TSD solution, which was stored at 4°C. Based on the conversion relationship between human and mouse body surface area and the results of different dose screening in the previous study (Xie et al., 2023), the equivalent dose was determined. A 10-time equivalent dose of TSD was selected as 66.3 g/(kg·d). Lastly, each mouse in the TSD group received TSD once a day, while the other group received normal saline.

#### Serum preparation

Animal experiments were conducted at the Experimental Animal Center of Huazhong Agricultural University (ethical number: 202209040003). To observe the responsiveness of the intestine to TCM under sterile and aseptic conditions, sterile mice were instilled with TCM in the TCM group (Experiment group), SPF mice were instilled with TCM in the TCM group (Control group), and SPF mice were instilled with saline in the saline group (Blank group) for 7 consecutive days, and blood was taken from the eyes 2 hours after the last instillation to prepare serum containing the drug. The serum of all mice in each group was mixed and clot for 1 h at room temperature. By centrifuging the supernatant at 4000 rpm for 10 minutes, serum samples were obtained. Serum samples were inactivated in a 56°C water bath for 30 minutes and filtered with 0.22um cellulose acetate membrane. Supernatants from serum samples were collected and stored at -80°C until analysis. To prepare a culture medium containing 10% drug serum, the processed serum was mixed with DMEM/F-12. To culture cells, the prepared medium was stored at 4°C.

#### LC-MS/MS analysis

Data analysis of metabolomics projects based on the LC-MS/MS system combined quadrupole Orbitrap mass spectrometer (Q Exactive Orbitrap, Thermo Fisher Scientific, USA). The underlying data analysis is univariate statistical analysis and multivariate statistical analysis (MVA) of the qualitative and quantitative results of the metabolome to screen for significantly different metabolites. )The Orbitrap platform is an electrospray ionization source with both positive ion mode (POS) and negative ion mode (NEG), and the combination of these two modes allows for better metabolite coverage in the detection of metabolomes.

#### Cell culture

U87–MG and U251-MG glioma cells (WHO IV, glioblastoma multiforme) were gifts from Dr. Shao (Nanjing Medical University, Nanjing, PRC). We maintained glioma cells at 37°C, 5% CO_2_ in 1:1 DMEM/F-12 with 10% FBS and 2 mm Gln, and used them in experiments within the 20 passages. For 24 hours, each group of cells was incubated with blank medium. Then the experiment group and control group cells were exposed to the replaced medium containing each drug serum for another 24 h. The blank group cells were exposed to the replaced medium containing blank group drug serum for another 24 h.

#### Cell colony formation assay

After treatment of mice serum, cells were dissociated and seeded in 3.5 cm dishes at a destiny of 1.0 × 103 cells/well and then cultured for 14 days. After being fixed with 4% formaldehyde for 15 minutes, the cells were stained with 0.1% crystal violet for 10 minutes. Counting the colonies was done after washing in PBS three times.

#### Cell proliferation assays

A cell proliferation assay (BeyoClick™ EdU-594 Cell Proliferation Kit, Beyotime, China) was used for the analysis according to the manufacturer’ s instructions. Using a Zeiss laser scanning microscope, EdU cells were imaged.

#### CCK8 assay

We used the CCK8 kit (Dojindo, Shanghai, China) to perform the assay. Each well was planted with 1,000 cells in 100 μl medium, and 10 μl CCK8 was added to each well. In a humidified incubator with 5% CO_2_, cells were incubated at 37°C for 24 hours to determine their proliferation ability.

#### RNA-seq

Each group of cells was in logarithmic growth phase and exposed to the respective group’s mouse serum medium for at least 24h. An RNA transcriptomic analysis was carried out by Shanghai OE Biotechnology Co., Ltd. In order to unravel the secondary structure of total RNA, it was diluted in diethylpyrocarbonate (DEPC) water and denatured at 65°C. Magnetic beads containing oligo(dt) were used to enrich mRNA, after which the mRNA was eluted using a heating method and fragmented. We synthesized the first strand of cDNA using random hexamers and mRNA as templates. After adding the buffer solution, dNTPs, RNase H, and DNA polymerase I, the second strand of cDNA was synthesized. Eluate the cDNA with the elution buffer after it has been purified using magnetic beads. Following end repair, cDNA libraries were sequenced using Illumina’s HiSeq™ 2000 system using the paired-end method.

#### Data sources

The Cancer Genome Atlas (TCGA) database (https://portal.gdc.cancer.gov/) provides a representation of cancer gene expression, miRNA expression, copy number variation, DNA methylation, and single nucleotide polymorphisms (SNPs). We retrieved mRNA expression data from the TCGA database for the glioma cohort.

#### Gene set enrichment analysis

The effect of each group on different gene sets was evaluated using GSEA. The differentially expressed genes of each group and gene set were ranked using this method. Following alignment to the ranked list, the running sum was calculated (Subramanian et al., 2005). The enrichment scores were then normalized to obtain the Normalized Enrichment Score (NES). In the meantime, the false discovery rate (FDR) was calculated, and an FDR of 0.25 indicated statistical significance.

#### Protein-protein interaction network and functional enrichment analysis

By removing duplicates from each group, total DEGs were used to construct the PPI network *via* the STRING database (von Mering et al., 2003), followed by Cytoscape software’s topological analysis (Shannon et al., 2003). To examine the biological functions of these DEGs, functional enrichment analysis was performed *via* DAVID database on Gene Ontology (GO) and Kyoto Encyclopedia of Genes and Genomes (KEGG) (Huang et al., 2007). Based on the adjusted P-value of 0.05, GO terms and KEGG signaling pathways were enriched, followed by visualization of the top 20 most significant GO terms.

#### Gene expression level and survival analysis

Clinical information and transcriptomic data of 169 glioma samples, 5 normal tissue samples, 224 grade2 glioma samples, 245 grade3 samples, and 168 grade4 glioma samples were collected from The Cancer Genome Atlas (TCGA) data portal (Ceccarelli et al., 2016). We collected 1157 healthy tissues and organs from The Genotype-Tissue Expression Project (GTEx) database, despite the lack of para-cancerous samples (Vivian et al., 2017). The Wilcoxon test was used to compare gene expression levels between tumors and normal tissues. According to gene expression values, each tumor sample was divided into high- and low-expression groups. The cox proportional hazard model was then used to evaluate the association between gene expression and overall survival in gliomas.

#### Western blot

We extracted protein from 35 mm dishes containing cells grown in the TRIzol method. To quantify the extracted protein, Bradford’s assay was used. For all detections, 50 µg of protein was loaded on 12.5% SDS-PAGE gels. A Western blot was performed in accordance with the protocol provided by Abcam. Incubation with appropriate primary antibodies was performed overnight at 4°C following blotting. The blots were then left untreated for 1 hour at room temperature before being incubated with HRP conjugated secondary antibodies. ECL reagent was used to visualize the blots.

#### Real-time quantitative PCR analysis

TRIzol was used to isolate total RNA from cultured cells. cDNA was then reverse transcribed *via* HiScript II Q RT SuperMix for qPCR kit (Vazyme, China).The ChamQ Universal SYBR qPCR Master Mix (Vazyme, China) was used to assess gene expression in real-time. This study used a wide array of primer pairs to detect gene expression relative to GAPDH. The primer pairs used are provided in supplementary information.

#### Animal experiments

Human glioblastoma cells U87MG were injected intracranially to establish an orthotopic brain glioblastoma xenograft. As a brief description, nude mice (Cavens-biogle, China) were anesthetized and positioned in a stereotaxic instrument (MEYUE, China). Afterwards, a 2 mm section of the skull was ground with a dental drill (MEYUE, China) until it was soft and translucent. In the following, 1.5×10^5^ U87MG tumor cells (in 3 µL) were injected into the frontal region of the cerebral cortex for 5 minutes. Mouse head skin was closed using SILK sutures (Ethicon, USA) after implantation. A subcutaneous injection of U87 cells (5.0×10^6^ cells in 100 µL of DMEM) was used to implant human glioma xenografts into 4-week-old nude mice (Cavens-biogle, China) (Gong et al., 2022).

### Statistical analysis

For all numerical data, standard deviations compared with control are expressed as the mean of three independent experiments. We performed all statistical analyses with SPSS 20.0 software (SPSS Inc., Chicago, IL, USA). A level of p-value <0.05 was considered significant in all statistical tests.

## Results

### Taohong Siwu Decoction pharmaceutical chemical composition analysis

**Figure 1:**
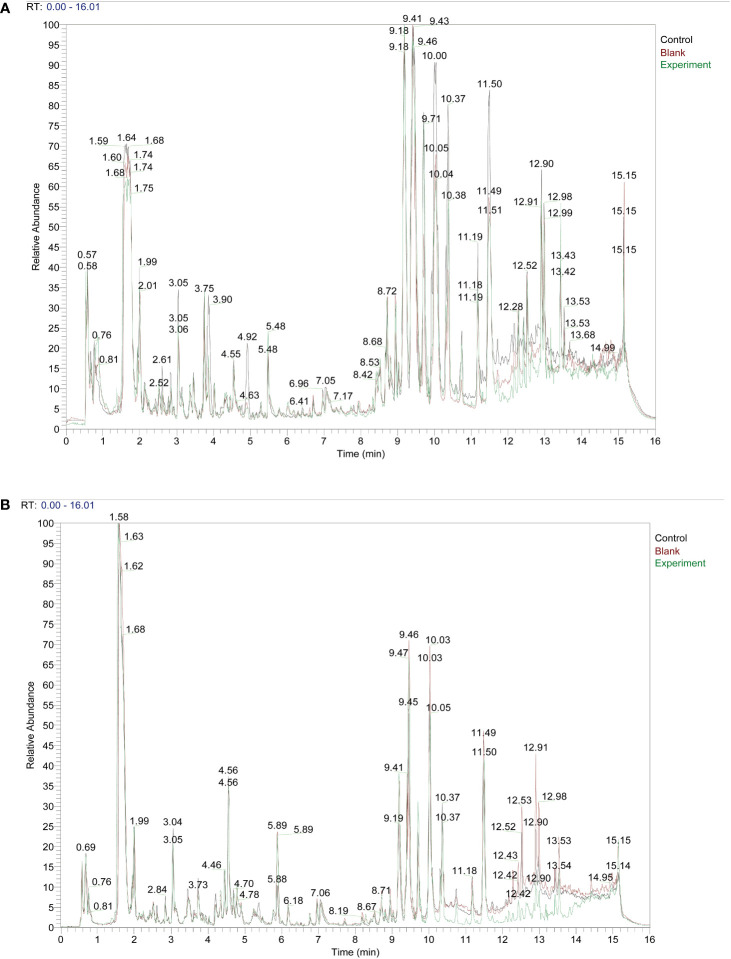
Taohong Siwu Decoction pharmaceutical chemical composition analysis: Experiment group showed the different TCM metabolites.) Control: serum from Taohong Siwu Decoction fed conventional SPF mice; Blank: serum from conventional SPF mice without Taohong Siwu Decoction feeding; Experiment: serum from Taohong Siwu Decoction fed Germ-free mice.). **(A)** Analysis of pharmaceutical serumcomposition in cationic mode of LC/MS; **(B)** Analysis of pharmaceutical serumcomposition in anionic mode of LC/MS.

We set up three subgroups Control group, Blank group, and Experiment group. The specific information is as follows: Control: serum from Taohong Siwu Decoction fed conventional SPF mice; Blank: serum from conventional SPF mice without Taohong Siwu Decoction feeding; Experiment: serum from Taohong Siwu Decoction fed Germ-free mice. We fed conventional SPF mice and Germ-free mice with Taohong Siwu Decoction and analyzed the drug composition in the serum of the two groups of mice (**Figures 1A, B**). To facilitate the follow-up study, we analyzed the serum pharmacological composition in both states to ensure quality control. The pictures revealed that the serum pharmacological composition in the two modes had significant differences, suggesting the necessity and feasibility of doing validation in subsequent experiments.

### Experimental flow diagram of serum from Taohong Siwu Decoction-fed mice interacting with glioma cells *in vitro*

**Figure 2:**
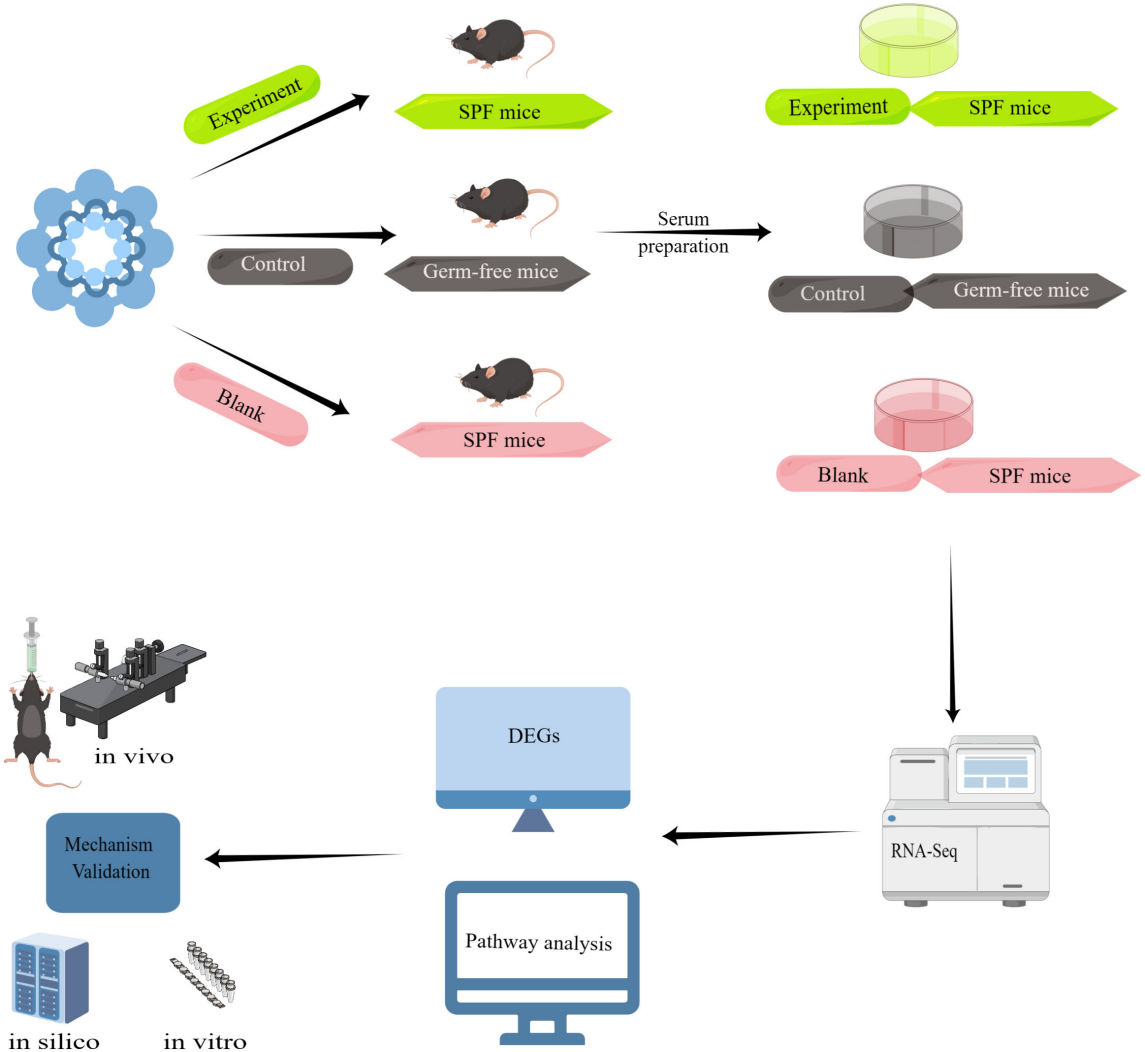
Experimental flow diagram: Taohong Siwu Decoction was used to feed SPF mice (Experiment) and germ-free mice (Control), and then co-cultured with serum and glioma cells from both groups. Then, we observed glioma cell phenotypes in the Experiment and Control groups. The serum of SPF mice without Taohong Siwu Decoction feeding was used for the Blank group. RNA-seq sequencing was performed on the cells of the three groups to compare the expression of differential genes. Bioinformatics was used for further analysis of the signaling pathways of differential gene expression. Q-PCR was performed to further validate the differential gene expression.

We divided the experiment into three major parts, the first part: Taohong Siwu Decoction was used to feed SPF mice (**Figure 2**) (Experiment) and germ-free mice (Control), and then co-cultured with serum and glioma cells from both groups; the second part: observation of glioma cell phenotypes in the Experiment and Control groups. The serum of SPF mice without Taohong Siwu Decoction feeding was used for the Blank group; Part III: RNA-seq sequencing was performed on the cells of the three groups to compare the expression of differential genes. Bioinformatics was used for further analysis of the signaling pathways of differential gene expression. Q-PCR was performed to further validate the differential gene expression.

### No phenotypic modulation of glioma cells by serum from Taohong Siwu Decoction-fed germ-free mice *in vitro*

**Figure 3:**
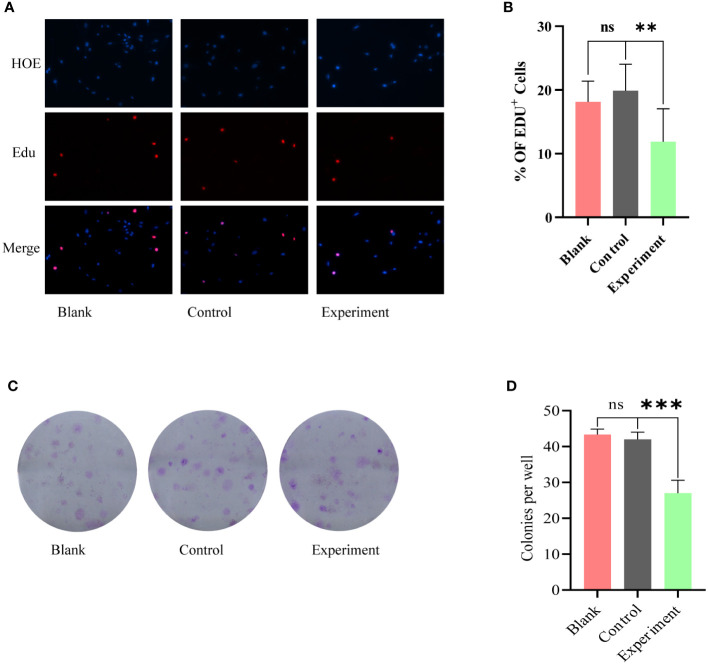
Phenotypic modulation of glioma cells by serum from Blank, Control, Experiment group: **(A)** EdU proliferation assay, no statistically significant difference between Blank and Control groups. The Experiment group showed a significant inhibition of proliferation. **(C)** Clone formation assay, the Experiment group exhibited a significant reduction in clone formation. **(B)** and **(D)** correspond to the results of the statistical analysis of **(A)** and **(C)**, respectively. ns, p≥0.05; **p ≤ 0.01;***p ≤ 0.001.

EdU proliferation assay and clone formation assay were used to further examine the co-cultured glioma cell lines. the results of EdU proliferation assay (**Figures 3A, B**) showed that there was no statistically significant difference between Blank and Control groups; the proliferation of glioma cells in Experiment group was inhibited. The results of clone formation assay (**Figures 3C, D**) were similar to the results of EdU assay. Among the three groups of cells, the Experiment group exhibited a significant reduction in clone formation, and this reduction was statistically different.

### Transcriptome differential gene screen suggests significant changes in CDC6 pathway activity

**Figure 4:**
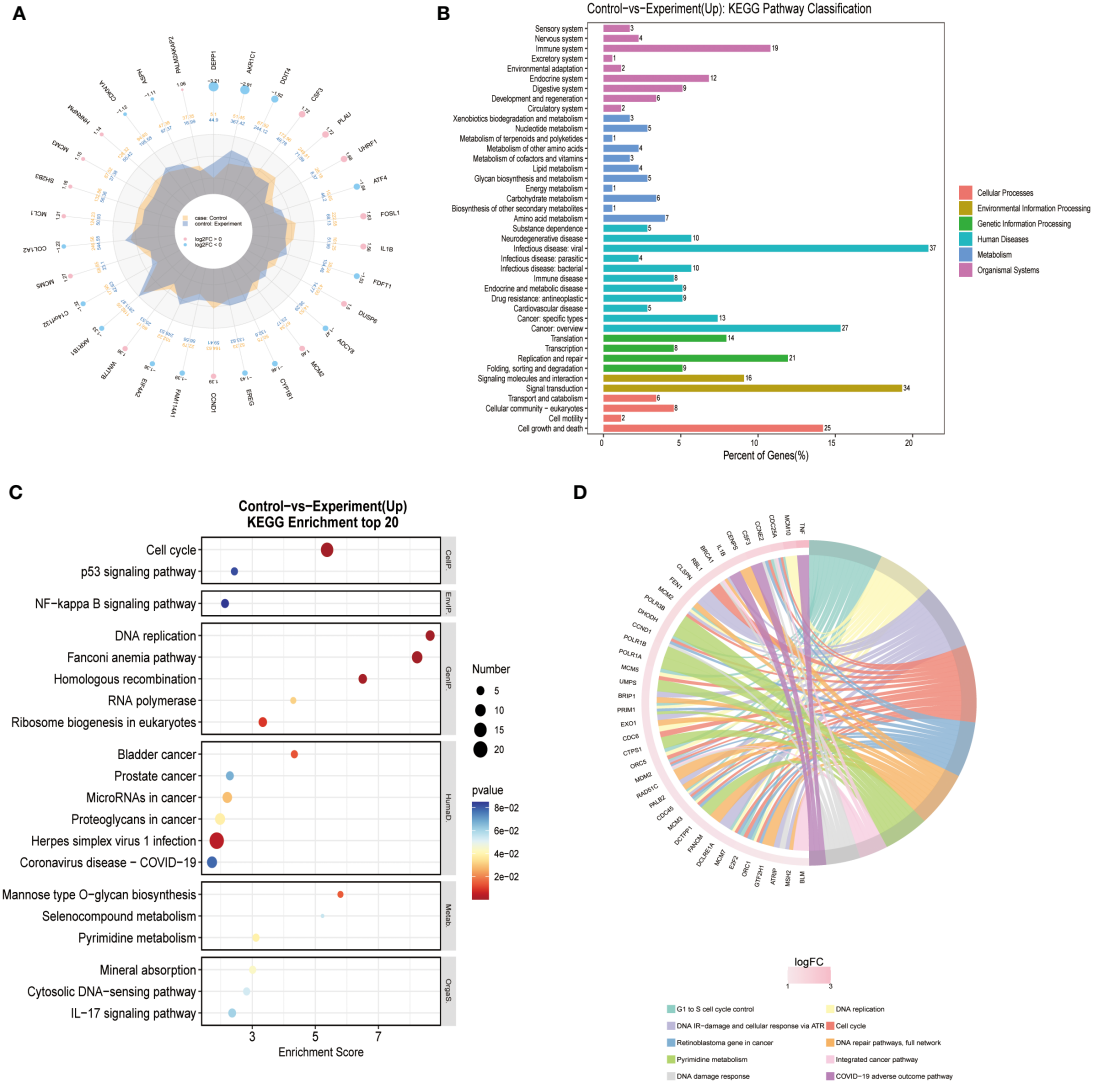
Transcriptome differential gene screen: **(A)** Different Expressed Gene (DEG) analysis was performed for Control and Experiment group. **(B)** KEGG pathway analysis showed that DNA replication pathway was essential for the difference of two groups. **(C)** KEGG enrichment of top 20 genes also confirmed that DNA replication pathway was the key point. **(D)** Reactome enrichment analysis also suggested that DNA replication pathway was valued for furthermore analysis.

We performed RNA sequencing on three different groups of cells (Blank/Control/Experiment). There was no significant difference in sequencing results between the Blank and Control groups. The differentially expressed genes were searched for and pathway analysis was performed. The results of the analysis showed that cells in the Experiment group showed the most significant differences in the pathway of DNA replication (**Figure 4**) the growth of glioma cells in the Experiment group was inhibited, most likely as a result of the inhibition of DNA replication.

### *In vitro* and vivo experimental validation of genes of proliferation pathway

**Figure 5:**
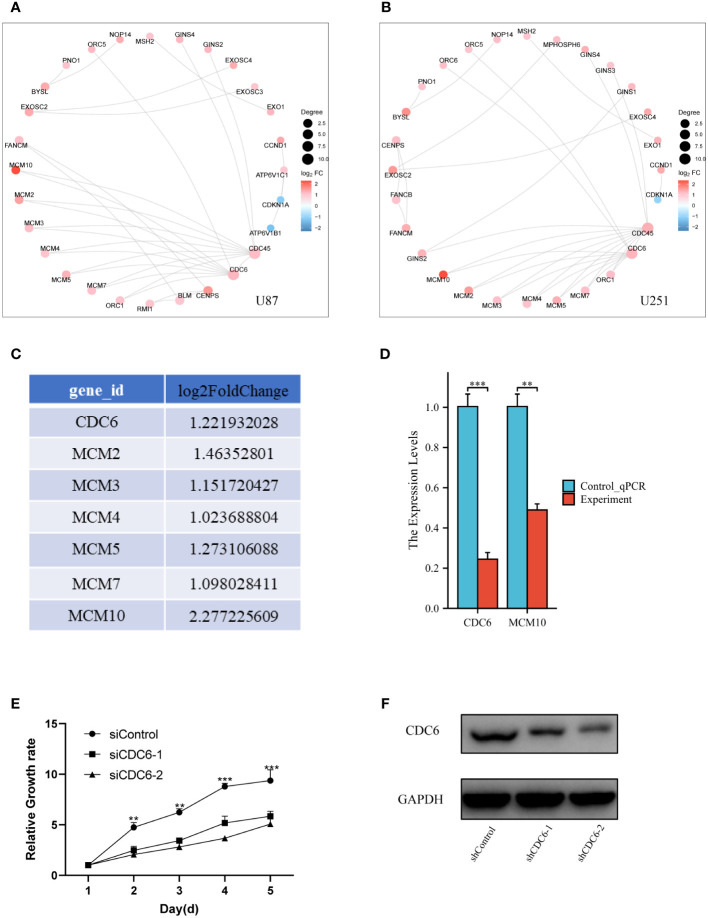
Validation of genes of proliferation pathway: **(A)** and **(B)** PPI network of different expressed genes, CDC6 was crucial for proliferation, which also had a significant change in bioinformatic analysis. **(C)** We select the CDC6 pathway genes and listed their log2FoldChange value. **(D)** Q-PCR was performed for CDC6 and MCM10. Both of two showed significant down-expression in Experiment group. **(E)** In U87 glioma cells, the proliferation of tumor cells was inhibited by the addition of siCDC6. **(F)** CDC6 expression was reduced in a subcutaneous glioma model in the shCDC6 group of mice. **p ≤ 0.01;***p ≤ 0.001.

Furthermore, more bioinformatic analysis was performed for different expressed genes in two different glioma cell lines. Protein-Protein Interaction (PPI) analysis was consistent with transcriptome different gene screening (**Figures 5A, B**). CDC6, an crucial gene for cell proliferation, was selected to analyzed it’s DNA replication pathway (**Figure 5C**). Then, Q-PCR confirmed that MCM 10 and CDC6 were high expressed in glioma cells of Control group (**Figure 5D**). Glioma proliferation was inhibited by knocking down CDC6 in U87 glioma cells by means of small interfering RNA (**Figure 5E**). CDC6 expression was evaluated (**Figure 5F**) in a mouse subcutaneous glioma model, and it was found that the shCDC6 group did express decreased CDC6. On this basis we evaluated whether glioma growth was restricted in the mouse *in situ* glioma model due to the low expression of CDC6. The results of the experiments we put in the supplementary material.

### CDC6’s validation of glioma in silico

**Figure 6:**
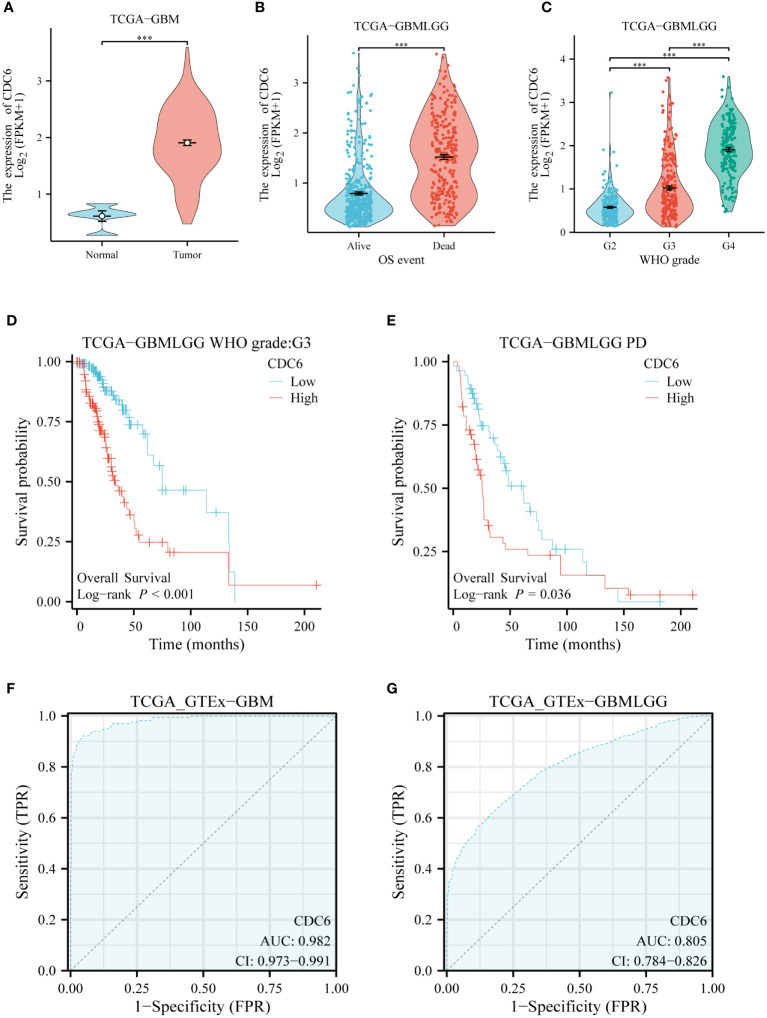
CDC6’s validation of glioma in silico: **(A)** CDC6 is highly expressed in gliomas. **(B)** High expression of CDC6 in glioma patients with short overall survival. **(C)** As the grade of glioma increased, CDC6 expression continued to rise and the difference was statistically significant. **(D)** In patients with grade III glioma, survival was shorter in patients with high CDC6 expression (p<0.001). **(E)** Survival was shorter in patients with progression despite treatment of low-grade gliomas with high expression of CDC6 (p<0.05). **(F)** and **(G)** show that CDC6 has a high TPR and FPR to distinguish tumor and normal tissues in GBM and low-grade glioma (LGG).

We evaluated the clinical relevance of CDC6 in gliomas using data from the TCGA database. CDC6 was highly expressed in patients with low-grade gliomas with short overall survival.CDC6 was highly expressed in gliomas and increased with increasing glioma grade (**Figures 6A–C**). Further, we evaluated the relationship between CDC6 expression and the prognosis of glioma patients. We found that patients with unremitting gliomas in grade III and low-grade gliomas were highly expressed in CDC6 and had shorter survival after treatment (p<0.05) (**Figures 6D, E**). Finally, we plotted the ROC curve of CDC6 as an independent indicator to determine normal and glioma tissues (**Figures 6F, G**).

### Diagram of gut microbiome regulated Taohong Siwu decoction metabolism on glioma cell phenotype *via* proliferation pathway

**Figure 7:**
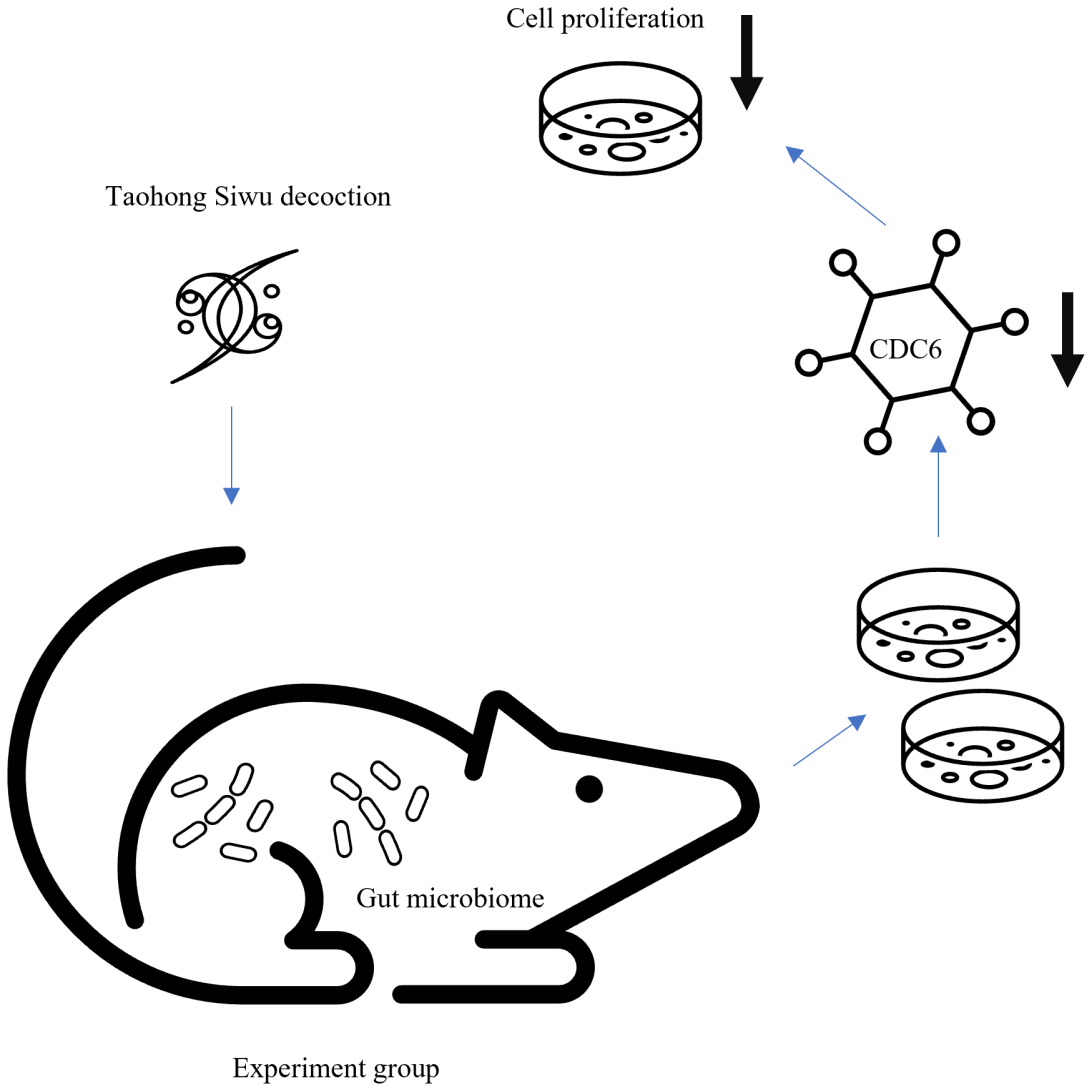
Diagram of gut microbiome regulated Taohong Siwu decoction metabolism: Taohong Siwu decoction metabolism was modulated by gut microbiome, yielding the active ingredient in serum from SPF mice. Then, this anti-tumor ingredient suppressed glioma cells *in vitro*.

We believed that Experiment group’s suppression of proliferation was due to expression of CDC6 (**Figure 7**). Gut microbiome modulated Taohong Siwu decoction metabolism and yielded the active ingredient in SPF mice serum. Then, this anti-tumor ingredient showed the suppression of glioma cells *in vitro*.

## Discussion

Gut microbiomes play a vital role in drug metabolism by directly interacting with drugs, altering their bioavailability, and contributing to their transformation in the body. The gut microbiota can influence drug metabolism through several mechanisms (Ianiro et al., 2016; Lange et al., 2016; Li et al., 2016; Weiss and Hennet, 2017; Azad et al., 2018; Wieers et al., 2019; Dikeocha et al., 2022), including altering drug absorption, modifying drug bioavailability, interfering with drug distribution, and influencing drug elimination. Intestinal bacteria can affect the absorption of drugs by modifying the gastrointestinal environment, such as pH and motility, and by interacting with drug transporters in the intestinal lining. Some gut bacteria can produce enzymes that modify drugs directly or indirectly by producing enzymes that hydrolyze prodrugs into their active forms. The gut microbiota can affect drug distribution by altering the protein binding of drugs, which can increase or decrease their availability to tissues. Intestinal bacteria can metabolize drugs into inactive metabolites that are eliminated through feces, and they can also affect the activity of drug-metabolizing enzymes in the liver. There have been several problems with previous studies investigating the relationship between gut microbiomes and tumors. The most prominent issue is a lack of standardization and causal relationship. There is currently no standardized methodology for characterizing the gut microbiota, making it difficult to compare results across different studies. Many studies have identified correlations between the gut microbiota and various health outcomes, but it can be challenging to determine whether these correlations are due to a causal relationship between the gut microbiota and the health outcome or whether they are simply coincidental.

Germ-free animals, which are animals that are raised in a sterile environment and lack any intestinal microbiota, have the advantage of studying the role of gut microbiota in human tumors (Grover and Kashyap, 2014). Germ-free animals allow researchers to study the effects of specific microbiota on host physiology by introducing defined microbial communities or specific microorganisms (Manca et al., 2020a). This level of control is not possible in animals with complex microbiota. Secondly, germ-free animals eliminate the influence of confounding variables, such as diet and environmental factors, that can impact the gut microbiota in animals with complex microbiota (Manca et al., 2020b). This allows researchers to isolate the effects of the gut microbiota on host physiology. Thirdly, germ-free animals provide a standardized experimental model that is highly reproducible, which facilitates comparisons across different studies (Paik et al., 2020). Also, germ-free animals have reduced animal-to-animal variability compared to animals with complex microbiota, which increases the statistical power of experiments (Wu et al., 2021). Above all, germ-free animals can help establish causal relationships between gut microbiota and host physiology, which is not possible in observational studies with animals with complex microbiota. Germ-free animals allow researchers to study the molecular mechanisms of host-microbiota interactions without interference from other microbial populations.

One of the difficulties in herbal medicine efficacy studies is that there are too many confounding factors, and it is not very clear what kind of ingredients work, and it is even more difficult to explore very clearly what molecular mechanisms work (Li and Zhang, 2013; Wang et al., 2021; Yang et al., 2022). We have done RNA-seq analysis to address this challenge. Interestingly, our results showed that TSD metabolized by the intestinal flora produced an active ingredient that effectively inhibited DNA replication. This result is also consistent with our *in vitro* cell phenotyping results. Further, by bioinformatic analysis, our targeted molecules to explain this phenomenon was targeted to CDC6. This was confirmed by *in vitro* Q-PCR experiments. The reason why the genes with the largest changes in logFC values were not chosen is that we preferentially compared differential signaling pathways analyzed possible protein regulatory networks and changes in logFC values, and combined with previous studies, we found significant changes in CDC6-related signaling pathways.

The CDC6 gene is a key gene in the regulation of the human cell cycle, and it plays an important role in DNA replication and cell division (Hofmann et al., 2011; Shi et al., 2021; Zhang et al., 2023). Recent studies have shown that the CDC6 gene plays an important role in a variety of tumors, including gliomas (Wang et al., 2022). Glioma is a common central nervous system tumor, and its pathogenesis is still not fully understood (Ludwig and Kornblum, 2017; Ni et al., 2023). However, several studies have identified that the CDC6 gene may play a key role in the development and progression of gliomas. For example, a study published in the journal Nature Communications found that CDC6 gene expression levels were significantly elevated in gliomas, and its overexpression was strongly associated with tumor malignancy and prognosis. Multiple types of tumors, including gliomas, were shown to express higher levels of CDC6. As well as age, IDH status, 1p/19q coding status, WHO classification, poor overall survival (OS), and histological type of glioma, high CDC6 expression was significantly associated with poor OS. This correlation of CDC6 with tumor cell proliferation was similarly observed in our experiments. This may also be the key to the ability of TSD to inhibit tumor proliferation. The primary function of CDC6 is to participate in DNA transcription and cytokine production pathways. As a result of gene set enrichment analysis (GSEA), it was revealed that patients with high CDC6 expression are differentially enriched for the MAPK pathway, the P53 pathway, and the NF-kappa B pathway. In gliomas, CDC6 expression was positively related to Th2 cells, macrophages, and eosinophils and negatively related to plasmacytoid dendritic cells and CD8 T cells, and NK CD56 bright cells. In addition, another study found that the growth and proliferation of glioma cells could be inhibited by interfering with the expression of the CDC6 gene. This suggests that the CDC6 gene may be a potential therapeutic target for gliomas.

Our study found a direct relationship between intestinal flora and DNA replication. Previous studies have mainly summarized the correlation between the two and did not directly observe experimental evidence. On the one hand, intestinal flora can influence the health of human cells. For example, several studies have shown that dysbiosis of the intestinal flora is associated with the development of several diseases, such as intestinal inflammation, autoimmune diseases, and obesity. The occurrence of these diseases may affect the DNA replication of human cells, which in turn affects human health. On the other hand, DNA replication in human cells can also affect the health of the intestinal flora. For example, errors in DNA replication may lead to cell mutations, which in turn may lead to the development of cancer. The growth and division of these cancer cells may affect the composition of the intestinal flora and thus lead to an imbalance in the intestinal flora. However, we clarified by RNA-seq technique that the intestinal flora metabolizes Taohong Siwu Decoction to produce an active citadel that inhibits DNA replication and thus regulates DNA replication. This also explains the causal relationship between the intestinal flora regulating the disease phenotype from another perspective. Taohong Siwu Decoction is a common herbal soup. It has been used in Chinese TCM clinics for thousands of years with definite efficacy, but the mechanism of onset of action is not well understood. We chose such a common herbal soup with definite clinical efficacy to exclude as many experimental interfering factors as possible.

Our findings validate the inferences of previous studies and clarify that intestinal flora is an integral part of drug efficacy. The reasons for clinical drug treatment failure in glioma may be directly related to intestinal flora. Serum from sterile animals after drug metabolism is an important tool to confirm the role of intestinal flora. This tool allows a series of patterned experiments to be performed to verify the interaction between intestinal flora and drug metabolism. The mechanism of TCM action has long been poorly understood, and it isn’t easy to elucidate the molecular mechanism using the existing body of research. Our study provides an alternative perspective on the possible mechanisms of TCM onset and a new way of investigating the efficacy of TCM, which has been clinically proven for thousands of years. Our study explored *in vitro* experiments using serum from sterile animals after metabolizing Taohong Siwu Decoction. RNA-seq was used to explore the modulatory effect of intestinal flora on the efficacy of Taohong Siwu Decoction. Further, we selected the most malignant glioma cell line for the validation of drug efficacy. The experimental results showed that Taohong Siwu Decoction metabolized by the intestinal flora suppresses the malignant phenotype of glioma cells. This also provides new ideas and methods for the study of TCM against tumors.

Of course, there are some shortcomings in our study. The targets of gut flora modulating drug efficacy are not fully defined. Large-scale population studies on the effect of intestinal flora on drug efficacy are lacking.

## Conclusions

Our study verified the direct modulatory effect of intestinal flora on drug treatment of tumors by serum from sterile animals after drug metabolism. We established a new method to quantify the relationship between intestinal flora and the regulation of TCM efficacy through this study.

## Data availability statement

The data presented in the study are deposited in the GSA repository, accession numberCRA011197, https://bigd.big.ac.cn/gsa/browse/CRA011197.

## Ethics statement

The animal study was reviewed and approved by Experimental Animal Center of Huazhong Agricultural University.

## Author contributions

SF: conceptualization, investigation, writing—original draft, formal analysis. QW: investigation, resources, methodology. CZ: writing—review, and editing. WW: methodology. conceptualization. HL: conceptualization, and resources. XL: writing—review and editing, supervision. All authors have read and agreed to the published version of the manuscript.
