# Supplementary material for: Effect of gut microbiome regulated Taohong Siwu Decoction metabolism on glioma cell phenotype
Source: Front Cell Infect Microbiol. 2023 Jun 5;13:1192589. doi: 10.3389/fcimb.2023.1192589 (PMC10277651; doi:10.3389/fcimb.2023.1192589)
Supplement: Supplementary file 2 [file Table_2.docx]

Supplementary Material

Effect of Gut Microbiome Regulated Taohong Siwu Decoction Metabolism on Glioma Cell Phenotype

**Suyin Feng ^1,2#^, Quan Wan^3#^, Weijiang Wu^1#^, Chenyang Zhang^2^, Hua Lu ^1*^, and Xiaojie Lu ^2,3*^**

*** Correspondence:** luhua1969@hotmail.com (H.L.); wxluxiaojie@outlook.com (X.L.)

# Supplementary Data

Supplementary Material should be uploaded separately on submission. Please include any supplementary data, figures and/or tables.

Supplementary material is not typeset so please ensure that all information is clearly presented, the appropriate caption is included in the file and not in the manuscript, and that the style conforms to the rest of the article.

# Supplementary Figures and Tables

## Supplementary Figures

**
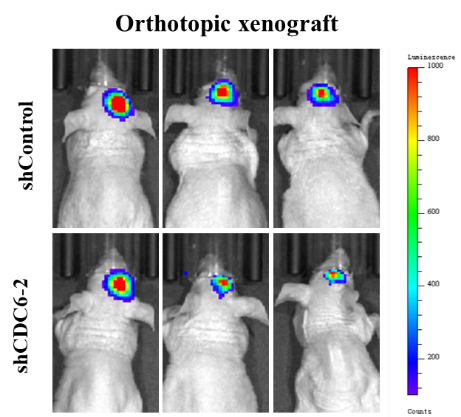
**

**Supplementary Figure 1.** We selected the more effective shCDC6-2 cell line to establish an in situ model of glioma in nude mice, and in situ tumorigenesis was inhibited in the shCDC6-2 group compared with the control group.

## Supplementary Tables

| Marker | Gene | TargetSeq | GC% |
| --- | --- | --- | --- |
| Target 1 | Cdc6 | GAGATCAGGTTCTGGACAA | 47.36 |
| Target 2 | Cdc6 | GGAGAGCTATTGAAATTGT | 36.84 |
| NC | Cdc6 | TTCTCCGAACGTGTCACGT | 52.63 |

**Supplementary Table 1.** Targeting Cdc6 siRNA sequences.

| Gene name | Primer sequence (5'-3') |
| --- | --- |
| *Beta-Actin* | Forward: CATGTACGTTGCTATCCAGGC |
|  | Reverse: CTCCTTAATGTCACGCACGAT |
| *CDC6* | Forward: TTTAGGTAGTGAGAAGGGGAATTATATT |
|  | Reverse: TAAAAAAACCCCAAATCTTAAAATC |
| *MCM10* | Forward: GAAGAAGGTTACGCCACAGAG |
|  | Reverse: TTTACAGGTTCCCAGGTCAAG |

**Supplementary Table 2.** Primer sequences used in RT-qPCR
